# Development of accreditation standards for interprofessional education: a Canadian Case Study

**DOI:** 10.1186/s12960-020-00551-2

**Published:** 2021-01-20

**Authors:** Ruby E. Grymonpre, Lesley Bainbridge, Louise Nasmith, Cynthia Baker

**Affiliations:** 1 College of Pharmacy, Rady Faculty of Health Sciences, University of Manitoba, 750 McDermot Ave, Winnipeg, MB R3E 0T5 Canada; 2 Department of Physical Therapy, Faculty of Medicine, University of British Columbia, Vancouver, BC Canada; 3 Associate Faculty, School of Leadership Studies, Royal Roads University, Victoria, BC Canada; 4 Department of Family Practice, University of British Columbia, Vancouver, BC Canada; 5 Canadian Association of Schools of Nursing and Professor Emeritus, Queens University, Kingston, ON Canada

**Keywords:** Accreditation, Interprofessional education, Health professional education

## Abstract

**Background:**

Academic institutions worldwide are embedding interprofessional education (IPE) into their health/social services education programs in response to global evidence that this leads to interprofessional collaborative practice (IPC). The World Health Organization (WHO) is holding its 193 member countries accountable for Indicator 3–06 (‘IPE Accreditation’) through its National Health Workforce Accounts. Despite the major influence of accreditation on the quality of health and social services education programs, little has been written about accreditation of IPE.

**Case study:**

Canada has been a global leader in IPE Accreditation. The Accreditation of Interprofessional Health Education (AIPHE) projects (2007–2011) involved a collaborative of eight Canadian organizations that accredit pre-licensure education for six health/social services professions. The AIPHE vision was for learners to develop the necessary knowledge, skills and attitudes to provide IPC through IPE. The aim of this paper is to share the Canadian Case Study including policy context, supporting theories, preconditions, logic model and evaluation findings to achieve the primary project deliverable, increased awareness of the need to embed IPE language into the accreditation standards for health and social services academic programs. Future research implications are also discussed.

**Conclusions:**

As a result of AIPHE, Canada is the only country in the world in which, for over a decade, a collective of participating health/social services accrediting organizations have been looking for evidence of IPE in the programs they accredit. This puts Canada in the unique position to now examine the downstream impacts of IPE accreditation.

## Background

Globally, evidence continues to emerge in support of interprofessional education (IPE) as the essential first step in developing the interprofessional collaborative capabilities required for the provision of collaborative person-centred practice (IPC). To be effective and meaningful, IPE requires more than just learners from different professions sitting together listening to the same lecture or in a one-way exchange of knowledge between two professions [1]. IPE is defined as ‘Occasions when members or students of two or more professions learn about, with and from each other, to improve collaboration, and the quality of care and services’ [2].

IPE is not a recent phenomenon. The assertion “*if the health professionals are to work together, they also must learn together*” was posed by George Szasz back in 1969 [3]. More recently, in its *Framework for Action on Interprofessional Education and Collaborative Practice* the World Health Organization (WHO) has acknowledged that *‘After almost 50 years of enquiry…there is sufficient evidence to indicate that effective interprofessional education enables effective collaborative practice’* and further notes that *‘collaborative practice strengthens health systems and improves health outcomes* [4]. The WHO Framework for Action calls for innovative, collaborative health and social service delivery models to address fragmented health systems, avoidable patient safety and quality of care issues, global shortages of health and social service providers, and unnecessary health service delivery costs. Although more research is needed, there is emerging evidence that the benefits of IPC include improved access to safe, quality care, reduced lengths of hospital stay, improved quality of life for patients and families, and improved recruitment and retention of health and social service providers [5]. IPC promises to positively impact at least two of the ‘triple billion targets’ outlined in the WHO Impact Framework: ‘*1 billion more people better protected from health emergencies and 1 billion more people enjoying better health and well-being’ *[6]. IPC is also highly relevant to the call for improved health system performance set out by WHO Universal Health Coverage (UHC) through the health systems policy area of ‘Service Delivery’ most notably ‘*expanding frontline services, particularly primary health care*’ and ‘*improving patient safety and quality of health services*’ [7].

 As the precursor to IPC, and despite its complexities and logistical challenges, many academic institutions worldwide are experimenting with innovative approaches to embed IPE into the curricula of their health and social services education programs [4, 8–11]. In one global IPE environmental scan targeting health professional student educators from WHO’s 193 Member States, 396 surveys from 41 members countries were completed. Only 15% reported no experience facilitating IPE. Although a majority of responses (91%) came from Canada, United Kingdom and United States of America, respondents also included those from South Africa, South Asia, sub-Saharan Africa, China and Middle East, Mexico, and Poland, suggesting a steady global uptake of IPE. The authors did however caution that *“significant efforts are required to ensure that IPE is designed, delivered and evaluated in keeping with internationally recognized best practice”* pointing to a potential role for accreditation as a curriculum quality assurance mechanism [12].

Accreditation is a process in which an education program is assessed against a set of national standards. It is an external process that also serves as an incentive for innovation [13–17]. Despite the major influence of accreditation on pre-licensure health and social services education programs, little has been written about accreditation of IPE. Accreditation of IPE is complex for several reasons. Firstly, successful implementation of IPE is complex, as is IPC, the desired outcome of IPE. The D’Amour Oandasan Framework illustrates the micro- meso- and macro- level factors and relationships that must be considered when implementing IPE and IPC within and between the Educational and Professional Systems [18]. The Framework emphasizes the complexity of interventions required for change management in both academic and practice environments. Of note, the Framework identifies integration of IPE standards into the accreditation programs of health and social care education programs as a ‘macro’ level influence on the educational system to achieve the desired ‘Health Professional Learner Outcomes’ (i.e. collaborative capabilities) in practice after graduation. Secondly, the process for approving changes to accreditation standards is long and complex. The education and service communities that need to be engaged are diverse with each sector having its own culture, operational processes, and standards. Before formal approval, the accrediting body must first develop the standards as well as a survey tool or consultation mechanism to examine IPE standards in academic programs. The standards must be tested in one or more academic programs or submitted to a systematic and widespread review process after which revisions are necessary to refine the process, standards and/or tools. Moreover, some accreditation programs are shared across national borders requiring even more complicated approval and testing processes. The lack of a shared mental model (common lexicon) among the various organizations also presents challenges to accrediting organizations in their efforts to develop a common IPE learning outcome framework [19].

While it may seem a long way from accreditation decisions to actual patient care, national accreditation programs offer a key mechanism for assuring that interprofessional education which leads to collaborative patient-centred care is incorporated. The Lancet Report underscored the significant role accreditation plays in changing health professional education in general and made specific reference to the importance of aligning accreditation standards with health care reform and social accountability priorities [20]. To achieve the third generation of health professional education reform proposed by the Lancet Commission, ‘stewardship mechanisms, including socially accountable accreditation’ were identified as one of four enabling actions. [20].

## Case presentation

The following Canadian case study describes how the Accreditation of Interprofessional Health Education (AIPHE) project achieved its primary deliverable, increased awareness by 8 accrediting organizations for 6 health professions of the need to embed IPE language into the accreditation standards.^1^ Specifically we discuss the policy context, AIPHE background, supporting theories, preconditions, logic model, and evaluation results of the AIPHE project.

### Policy context

With concerns regarding the affordability and sustainability of the Canadian Health System, in 2001 the federal government established the Commission on the Future of Health Care in Canada. The mandate of the Commission was to engage Canadians in a national dialogue on the future of heath care and to make recommendations to preserve the long-term sustainability of Canada's universally accessible, publicly funded health care system. In his final report entitled: “Building on Values: The Future of Health Care in Canada” Commissioner Roy Romanow underscored the need for a coordinated approach to Health Human Resources planning and echoed the assertion made by George Szasz 30 years prior: *“If health care providers are expected to work together and share expertise in a team environment, it makes sense that their education and training should prepare them for this type of working arrangement"* thereafter referred to as Interprofessional Education (IPE) (p, 109) and further *“…the direction of our health care system must be shaped around health needs of individual patients, their families and communities”* thereafter referred to as Collaborative Person Centred Practice (CPCP) (p.50) [21]. As a direct consequence of this report and through its Pan-Canadian Health Human Resource Strategy, Health Canada funded the Interprofessional Education for Collaborative Person Centred Practice (IECPCP) Initiative. The goal of this 10 year $21 million investment was to support the development and implementation of innovative approaches to IECPCP.

### Accreditation of Interprofessional Health Education (AIPHE) project

Recognizing the strong influence that accreditation has on health professional education, one of the foundational projects funded through the IECPCP Initiative was an environmental scan and key informant interviews to understand the accreditation processes for 8 accrediting organizations of 6 health and social service professions – medicine, nursing, social work, pharmacy, physical therapy and occupational therapy [17]. A notable study finding was that although IECPCP was deemed to be important by all professions, few had explicit accreditation standards or criteria that promoted or fostered this new way of learning. At the time of the report, pharmacy was the only accreditation organization that made direct reference to IPE in its standards while the other five disciplines made only indirect references such as students functioning in complex environments and learning to communicate with health team members to achieve interdisciplinary collaboration. The authors also concluded that the main barriers to collaboration around standards for IPE included a lack of knowledge about other professions’ accreditation process and standards, stereotypes, and a lack of respect for other professions. Enablers for collaboration included existing collaborative structures, the need to keep pace with changes in the health care system, and supportive attitudes towards collaborative education and practice within accrediting organizations. Recommendations in the report included the need to: (1) explore and encourage joint IECPCP standards across the accrediting organizations; (2) engage key accreditation stakeholders in further discussions on the role of accreditation in supporting and fostering IECPCP; and (3) facilitate education and information-sharing among accrediting organizations.

Stemming from the recommendations of this study, Health Canada funded the two-phase (Phase 1 Oct 2007-Mar 2009; Phase 2 May 2010-Mar 2011) AIPHE project [1, 22]. AIPHE was a collaborative of eight national organizations that accredit pre-licensure education for the six Canadian health professions involved in the environmental scan. The vision of the AIPHE project was to promote interprofessional health professional education in order to develop a health care workforce capable of providing collaborative person-centred care in Canada.

A first step to AIPHE Phase 1 was to conduct a follow-up environmental scan and key informant interviews building on and using similar methodologies to the 2005 study [23]. Consistent with the former study, the follow-up scan indicated that only pharmacy accreditation standards explicitly addressed IPE. Although the accreditation standards for medicine, social work, physiotherapy and occupational therapy made reference to interprofessional collaboration as an important component of professional practice, they remained ‘silent’ on the concept of interprofessional education. The authors cautioned that such ‘silence’ could undermine the importance of interprofessional education in achieving interprofessional collaborative capabilities. There was, however, high level support by all accrediting organizations for the development of principles and standards for IPE across the professions.

### Theories underlying desired change

The desired change that AIPHE set as its goal was to increase awareness of the need to embed IPE language into the accreditation standards for health and social services academic programs. AIPHE methodologies were supported by knowledge transfer, attitude change (balance and congruity), and diffusion of innovation theories.

Knowledge transfer theory was relevant to AIPHE as there existed a knowledge-to-action gap between the emerging evidence supporting accreditation of IPE and policy decisions relevant to health professions’ education and practice. The five elements of Lavis’ Knowledge Transfer Framework include: What should be transferred to decision makers? To whom should research knowledge be transferred? By whom? How? With what effect?) [24]. Table 1 outlines how the AIPHE communications strategy aligned with all five elements this framework.

**Table 1:** AIPHE Communications strategy: Terms of Engagement^a^

Lavis’ Knowledge Translation Framework [24]	AIPHE communications approaches and strategies
What should be transferred to decision makers? With what effect?	Communications Objectives: Introduce IPE standards into health education accreditation
	Key messaging—Position phrases/messaging on the value of IPE accreditation that can be used in communications materials • Commitment of AIPHE partners to incorporate shared principles for IPE into accreditation standards • Work of AIPHE will contribute to learners who possess the knowledge, attitudes and skills needed to practice collaborative person-centred care • Long term goal to foster person centred model of care through collaborative service delivery
To whom should research knowledge be transferred?	Target Audiences—Accreditation of IPE is not be possible without the support of those who influence both education and practice settings: • Associations and accrediting organizations for the 6 participating health professions; their provincial and federal licensing authorities • Academic partners including faculty, their leaders, clinical educators and community-based preceptors • Health service managers for the learners’ clinical sites • Learners • Patients/service users Secondary audiences: • Hospitals and health authorities • Provincial Ministries of Health and senior post-secondary education ministry policy officials • Regulated and accredited Canadian health professions, with emphasis on those who share an interest in IPE accreditation • Health Canada
By whom?	Communications champions: • Position AIPHE Steering Committee as ambassadors • IPE health leads in university across the country can help with dissemination of information
How?	Approaches to achieving communications objectives—Generate interest and awareness • Engage partners and stakeholders • Disseminate information and obtain feedback • Create a vibrant online community • Web-based platform • Face-to-face meetings/gatherings and webinars
	Tactics—Approaches and activities/events outlined to enable strategies • Regular outreach and contact • Toolkit that communications champions can use to share information (Power Point presentation, key messages, question and answer document, fact sheet)
	Positioning—How the information being developed and implemented is beneficial to the professions involved • Medical education is undergoing change in Canada. There are synergies between this and AIPHE’s work that can be a major driver for IPE implementation
	The current environment and how it impacts on communication processes • Regularly inform target audiences on matters that may affect them, their policies, standards, processes
	Timing—Gant chart
With what effect?	Measurement and Evaluation—Evaluating the strategies and tactics, once implemented • Hiring Evaluation consultants
	Sustainability—Approaches to sustain AIPHE communications • Utilizing technology

^a^ Saunders N’Daw, A. Terms of Engagement: Outreach, Share, Connect – Communications strategy for AIPHE: Implementation and Sustainability. Fall 2010

Balance theory predicts that communications and a sharing of ideas among stakeholders would foster harmony in thoughts, positive emotions and a mutual acceptance of ideas [25]. Early in the project, AIPHE stakeholders may have had either negative or positive attitudes towards accreditation of IPE; such an imbalance motivates individuals to change. To achieve a positive change in attitudes, AIPHE communications facilitated interactions among stakeholders through, for example, teleconferences and face-to-face and virtual (webinar) meetings. Congruity theory suggests that attitude change is more challenging when competing cognitive elements exist and when individuals’ opinions around these elements are discordant [26]. In the case of AIPHE, stakeholders may have supported IPE but not accreditation or vice versa. Consistent with congruity theory, to increase the likelihood of changing attitudes in the minds of our stakeholders, AIPHE communications strategy placed equal value on both elements.

Greenhalgh’s Diffusion of Innovation Theory was also relevant to AIPHE [27]. According to this theory, the rate with which an innovation is adopted is dependent on a complex interplay of individual/organizational and internal/external influences. Most relevant to AIPHE, communication and influence, the role of expert opinion, champions, boundary spanners, change agents and social networking played an important role in spreading and sustaining AIPHE goals and objectives.

### Preconditions to desired change

**Fig 1.**
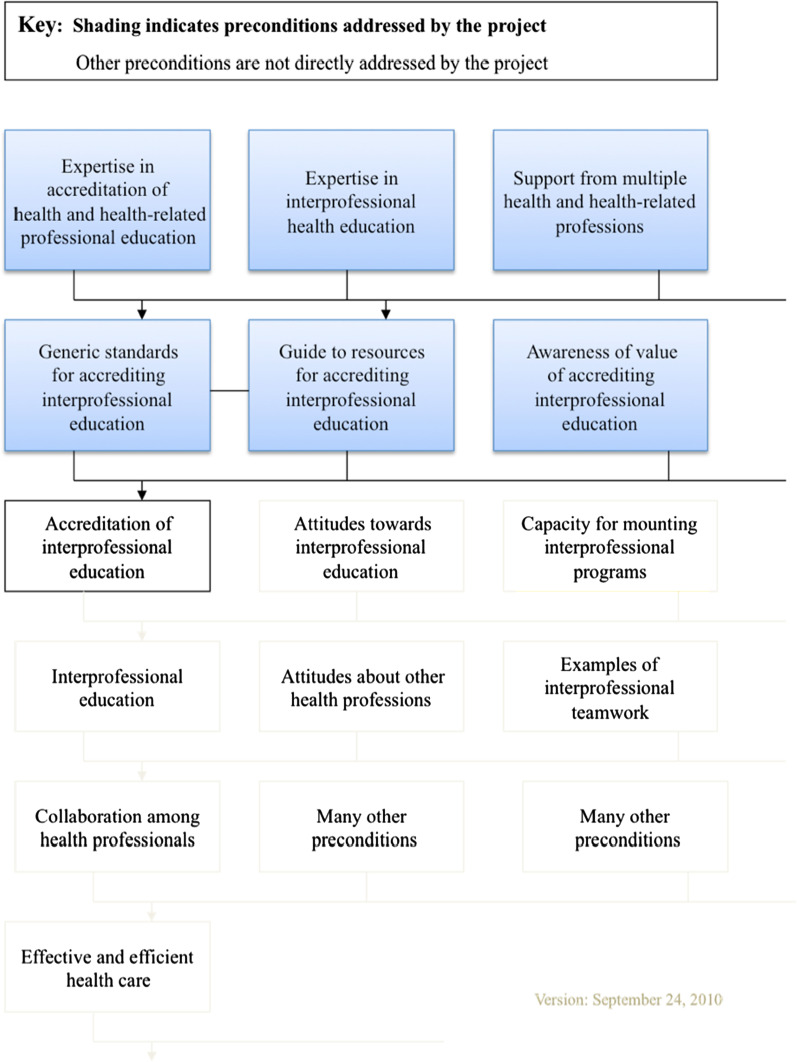
Preconditions model for the AIPHE project. Source: Intelligence Flows. Evaluation and Performance Measurement Framework for Phase 2 of the AIPHE Project. Oct 15, 2010. https://drive.google.com/file/d/1H9gAtOkbvNvZb7P5oobIHmGAeXFkw0Yb/view?usp=sharing

Over AIPHE Phases 1 and 2, two levels of pre-conditions needed to be addressed. The first level of preconditions included ‘expertise in accreditation of health and health-related professional education’, ‘expertise in interprofessional health education’, and ‘support from multiple health and health-related professions’. (Fig. 1) These preconditions were met during Phase 1 as a secretariat and committees worked collectively to build collaborative relationships and reach consensus on joint core accreditation principles. The Association of Faculties of Medicine of Canada (AFMC) served as the secretariat providing project management and administrative support. A Steering Committee with representation from the eight national accrediting organizations provided project oversight. Other Phase 1 participants included a 17 member Advisory Group with representation mostly from academia, Accreditation Canada to offer perspectives on IPE in practice environments and two Interprofessional Educators. In meeting these first level preconditions, the most significant tangible deliverable was the document entitled “AIPHE: Principles & Practices Implementation Guide” [1]. The guide provides an operational definition of IPE, guiding principles for integrating IPE standards into professional education, exemplars of standards, criteria, and evidence, a glossary of terms, and links to resources that would assist in curricular reform. The Guide was launched at a National Forum held on February 17, 2009 attended by 53 invited participants affiliated with a national organization and who were deemed to have influence in getting standards into accrediting structures and processes and/or had a desire to help to build capacity.

Phase 2 built on this foundational work and the growing momentum from Phase 1 and addressed the second level of preconditions: ‘generic standards for accrediting organizations’, ‘guide to resources for accrediting IPE’ and ‘awareness of the value of accrediting IPE’. A Standards Development Working Group (SDWG) was appointed from the 17 member Steering Committee with representation from the eight accrediting organizations. The mandate of the SDWG was to develop sample standards language. 

The Standards Development Working Group agreed to frame IPE relevant accreditation standards and criteria around 5 common domains: Organizational Commitment, Faculty, Students, Educational Program (Curriculum), and Resources. The Phase 2 publication entitled: “AIPHE: Interprofessional Health Education Accreditation Standards Guide” [22] suggests a range of options for standards language across all five domains as well as potential criteria and examples of evidence that could be used to demonstrate the level of compliance with the standards. The second level precondition ‘awareness of the value of accrediting IPE’ was also achieved through a cross-Canada knowledge exchange webinar that took place in November, 2010 bringing together 181 educators, clinicians, regulators, government officials, representatives of professional organizations and others across 14 sites to discuss a preliminary draft of the standards as well as an end-of-grant knowledge exchange face-to-face workshop that took place in March, 2011 involving members of AIPHE and representatives from over 26 other health profession accrediting organizations to explore and discuss lessons learned and challenges. This approach allowed sharing of information across a diverse group of participants and organizations/sectors, and in so doing facilitated collaboration and common understanding among the accrediting organizations.

### Logic model

**Fig 2.**
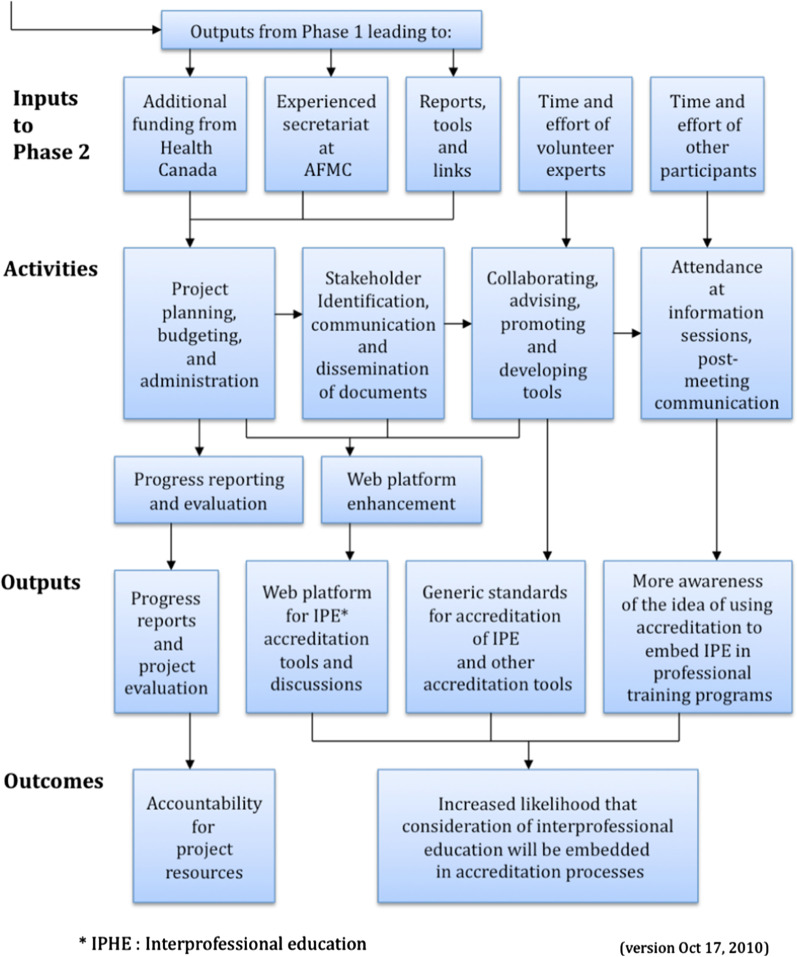
Logic model for phase 2 of the AIPHE project. Source: Intelligence Flows. Evaluation and Performance Measurement Framework for Phase 2 of the AIPHE Project. Oct 15, 2010. https://drive.google.com/file/d/1H9gAtOkbvNvZb7P5oobIHmGAeXFkw0Yb/view?usp=sharing

As part of AIPHE’s accountability to Health Canada, project evaluation included a logic model. (Fig. 2) A logic model is a visual reference of the inputs, activities, outputs and outcomes for a project. Inputs to Phase 2 included additional funding from Health Canada, the momentum and knowledge gained through Phase 1, an experienced secretariat, and the time and effort of volunteer experts and other participants. Project activities included stakeholder engagement through the November 2010 webinar and March 2011 knowledge exchange event, tool development and attendance at meetings. Outputs included project and evaluation reports, the Phase 2 Accreditation Standards Guide and raised awareness of the concept of accreditation of IPE. Project outcomes included accountability to the granting agency Health Canada, and the a priori desired change of AIPHE, increased consideration of IPE language embedded in accreditation processes.

### Project evaluation

An external consultant team was contracted to conduct the project evaluation. Methodologies included direct observation, on-line surveys and semi-structured interviews [28]. On-line surveys were sent to 181 individuals who attended the November 2010 webinar (50% response rate) and 20 individuals who attended the March 2011 knowledge exchange event. Twenty-one structured interviews were conducted with participating national accrediting organizations, management committee members, project staff and representatives from the 2011 knowledge exchange event. Findings were framed around five parameters: incremental effect of funding, tangible outputs, sustainability and momentum, unanticipated impacts, and lessons learned.

Project evaluators concluded that AIPHE Phase 2 achieved its overall goal: to increase awareness of the need to embed IPE language into the accreditation standards for health and social services academic programs. Findings suggest that all eight participating accrediting organizations indicated their intentions to embed IPE in their accreditation processes. Despite this strong momentum for change and desire for ongoing collaboration, respondents also expressed concern that there was still a long way to go before the concept of accreditation of IPE became mainstream; without a coordinating centre sustainability may be threatened and accrediting organizations may regress back to their uni-professional ways.

To understand the current state, the co-authors to this manuscript reviewed the most recent version of accreditation standards for each of the 8 accrediting organizations that participated in the AIPHE projects and extracted IPE relevant text. These data were tabulated against IPE relevant text extracted from the accreditation standards that were in place in 2005 (Additional file 1: Appendix S1). [29] A separate component of the project evaluation was a request to the 8 participating accrediting organizations to describe how they have embedded IPE language into their accreditation standards. The accrediting organizations prepared narratives, first in advance of the AIPHE Phase 2 knowledge exchange event (March 1, 2011) and a second time in advance of a Canadian Institute of Health Research (CIHR) funded Meeting and Planning event (Sept 10–13, 2012). Guided by the current state table, the research team also updated the 2012 narratives describing the progress of each participating accrediting organization in embedding IPE language into their standards. These updated narratives were subsequently reviewed and approved by each respective accrediting organization (Additional file 2: Appendix S2). [39].

With the exception of Pharmacy that already had IPE language in their accreditation standards prior to AIPHE, the other seven accrediting organizations progressed from making a verbal commitment through their participation in the AIPHE projects to ultimately embedding IPE language within their accreditation standards. In keeping with the initial AIPHE philosophy of flexibility and adaptability, the emerging standards and criteria look different across professions. Some programs embedded interprofessional education language across all domains including faculty, students, resources and curriculum or academic program while others focused primarily on the curriculum/academic program. Organizations are also at varying stages of implementation with some articulating explicit IPE standards/criteria and actively seeking evidence of IPE during their site visits while others are slowly developing, testing and integrating IPE.

## Discussion and conclusions

This work is highly relevant to the National Health Workforce Accounts explicit standard for IPE accreditation, specifically Standard 3–06 and its corresponding indicator “*Existence of national and/or subnational standards for interprofessional education in accreditation*” [40]. In addition to Canada, many other countries are in the preliminary stages of developing accreditation standards for interprofessional education. In the US, the Health Professions Accreditors Collaborative (HPAC) recently prepared a consensus guide endorsed by 24 HPAC member accrediting organizations to support a collaborative approach between educators and accreditors to develop, implement and evaluate innovative IPE approaches and IPE standards. [41] In Australia, the Health Professions Accreditation Collaborative Forum was established to foster collaborative approaches to accreditation, including ‘interprofessional education, learning, and practice.’ [42].

Other countries have some way to go in meeting the National Health Workforce Accounts Standard 3–06. Interprofessional.Global with its mission to “*serve as agents of change in providing global leadership to advocate for, collaborate on, promote, develop, and research IPECP innovation*” [43] has an important role to facilitate global uptake of accreditation of IPE. The Interprofessional.Global Policy Working Group is currently conducting a global scan of IPE Accreditation Standards. Preliminary findings suggest inconsistencies in accreditation across the globe; many countries, especially from lower income countries, do not require ‘accreditation’ of their health science education programs; other countries use varying terminologies such as ‘national evaluation’ and ‘common learning outcomes’; while others differ in how accreditation is conducted (e.g. by government, professional associations, private organizations). These finding are consistent with WHO recommendations around the impact of accreditation (strong recommendation) and IPE (conditional recommendation) on the relevance and quality of the health workforce [44]. Achieving global consensus on requirements for accreditation and accreditation of IPE is an essential consideration is transforming and scaling up health professions’ education and training.

In summary, interprofessional education is complex, interprofessional collaboration is complex, evaluation of quality and impact of IPE through accreditation is complex, and building collaborative relationships across sectors affected by accreditation adds to this complexity. In the Canadian case study, notwithstanding the changes made to accreditation standards across the 6 professions between 2005 and the present, there remain unanswered questions. While there is emerging evidence that IPE does positively influence collaborative practice and, de facto, health outcomes, the influence of IPE accreditation standards on the practice of IPE and its influence on collaborative practice and thus on practice environments or patient outcomes is still not empirically clear. IPE research has been criticized for being ‘values based’ as opposed to ‘outcomes based’ and lacking a theoretical basis [45]. IPE studies are classically short term, pre- / post-, self-reported student satisfaction measures of the IP event by students. A gap exists in understanding how the structures and processes of IPE translate into collaborative practice; the use of mixed methods drawn from the social sciences would provide more fulsome data.

With explicit IPE language in the accreditation standards across 6 health professions, Canada is now well positioned to advance to a higher level of research related to the implementation, evaluation and impact of the emerging IPE accreditation standards as an essential first step to graduating collaboration-ready health and social service professionals. The CIHC Accreditation Working Group is currently addressing this gap through a survey of Academic Programs to understand the evidence provided, exemplars and challenges in meeting IPE relevant accreditation standards. The alignment between the WHO and the global IPECP community also promises to catalyze recognition of the value accreditation of IPE across its 193 member states and advance research in this area.

## Supplementary Information

**Additional file 1.** Emergence of IPE Accreditation Standards within 8 accrediting organizations.

**Additional file 2. **Narratives outlining progress in accreditation of IPE across the health/social service professions involved in AIPHE.

## Data Availability

The datasets generated and analysed as part of this case study, specifically (Additional file 1: Appendix S1) entitled: Emergence of IPE accreditation standards within 8 accrediting organizations and (Additional file 1: Appendix S2) entitled: Narratives outlining progress in accreditation of IPE across the health/social service professions involved in AIPHE are available through the Canadian Interprofessional Health Collaborative (CIHC) website [29, 39].

## Abbreviations

AIPHE: The Accreditation of Interprofessional Health Education project
CACMS: Committee on Accreditation of Canadian Medical Schools
CASWE-ACFTS: Canadian Association for Social Work Education-Association Canadienne pour la Formation en Travail Social
CAOT: Canadian Association of Occupational Therapists
CASN: Canadian Association of Schools of Nursing
CCAPP: Canadian Council for the Accreditation of Pharmacy Programs
CIHC: Canadian Interprofessional Health Collaborative
CFPC: College of Family Physicians of Canada
CIHR: Canadian Institute for Health Research
CMQ: Collège des médecins du Québec
IECPCP: Interprofessional Education for Collaborative Person Centred Practice
IPC: Interprofessional Collaborative Practice
IPE: Interprofessional Education
LCME: Liaison Committee on Medical Education
PEAC: Physiotherapy Education Accreditation Canada
RCPSC: Royal College of Physicians and Surgeons of Canada
UHC: Universal Health Coverage
WHO: The World Health Organization

## Footnotes

1 The 8 accrediting organizations that formed the core of the AIPHE project included Physiotherapy Education Accreditation Canada, the Canadian Association of Occupational Therapists, the Canadian Council for Accreditation of Pharmacy Programs, the Canadian Association of Schools of Nursing, the Canadian Association for Social Work Education, the College of Family Physicians of Canada, the Committee on Accreditation of Canadian Medical Schools and the Royal College of Physicians and Surgeons of Canada.
