# Supplementary material for: Development of accreditation standards for interprofessional education: a Canadian Case Study
Source: Hum Resour Health. 2021 Jan 20;19:12. doi: 10.1186/s12960-020-00551-2 (PMC7818738; doi:10.1186/s12960-020-00551-2)
Supplement: Supplementary file 1 — Additional file 1. Emergence of IPE Accreditation Standards within 8 accrediting organizations. [file 12960_2020_551_MOESM1_ESM.pdf]

Appendix 1

Emergence of IPE Accreditation Standards within 8 accrediting organizations

(Between 2005 and the most recent version of respective uniprofessional Accreditation Standards Documents)

**Sample Uniprofessional Accreditation Standards relevant to IPE**	
**Undergraduate Medicine –**<br>Committee on Accreditation of Canadian Medical Schools (CACMS) in partnership with the Liaison Committee on Medical Education (LCME)	
Source: Curran 2005, Table 1, page 39	**Source:** Committee on Accreditation of Canadian Medical Schools (CACMS) Standards and Elements, 2019[30]
**ED-19**. There must be specific instruction in communication skills as they relate to physician responsibilities, including communication with patients, families, colleagues, and other health professionals.	**<u>Standard 7: Curricular Content</u>**<br>**7.8 Communication Skills**<br>The faculty of a medical school ensure that the medical curriculum includes specific instruction in communication skills as they relate to communication with patients and their families, colleagues, and other health professionals.<br>**7.9 Interprofessional Collaborative Skills**<br>The faculty of a medical school ensure that the core curriculum prepares

	medical students to function collaboratively on health care teams that include health professionals from other disciplines as they provide coordinated services to patients. These required curricular experiences include practitioners and/or students from the other health professions.
**Post Graduate Medicine – Family Medicine -** College of Family Physicians of Canada (CFPC)	
Source: Curran 2005, Table 2, page 45-46	Source: College of Family Physicians of Canada Standards of Accreditation for Residency Programs in Family Medicine, July, 2018[31]
Source: Curran 2005, Table 2, page 45-46	**Domain: Program Organization**<br>**Requirement 2.1.2** There are effective mechanisms to collaborate with the division/department, other programs and the post graduate office.<br>**Indicator 2.1.2.4:** There is collaboration with other health professions to provide educational experiences for learners across the spectrum of health professions.<br>**Domain: Education Program**<br>**Indicator 3.1.1.2:** The competencies address each of the Roles in the

	CanMEDS-FM Framework.<br><br>**CanMEDS/CanMEDS-FM** defines **‘interprofessional’** – Individuals from two or more professions (e.g. medicine and nursing) working collaboratively with shared objectives, decision-making responsibilities, and power to develop care plans and make decisions about patient care”[32]<br><br>**Domain: Resources**<br><br>**Indicator 4.1.2.3:** Resident training takes place in functionally inter- and intra-professional learning environments that prepare residents for collaborative practice.
**Post Graduate Medicine – Residency Programs** Royal College of Physicians and Surgeons (RCPSC) and College of Family Physicians of Canada (CFPC)	
Source: Curran 2005, Table 2, page 49-51	Source: Canadian Residency Accreditation Consortium: Excellence in Residency Accreditation: Standards of Accreditation for Residency Programs Version 1.2, 2018[33]
**Standard B.2: Goals and Objectives**	**Domain: Program Organization**

Goals and objectives should be structured to reflect and encourage the development of the CanMEDS roles:<br>Communicator: …discuss appropriate information with patients/families and the health care team…<br>Collaborator:…consult effectively with other physicians and health care professionals…contribute effectively to other interdisciplinary team activities . (p. 49-50)<br>**Standard B.5 Clinical, Academic and Scholarly** Content of the Program<br>2. Communicator<br>2.1 The program must ensure the residents learn effective communication skills for:<br>2.1.1 interacting with patients and their families,	**2.1.2.4** [Exemplary]: There is collaboration with other health professions to provide innovative educational experiences for learners across the spectrum of health professions. (p. 8)<br>**Domain: Education Program**<br>**3.1.1.2**: The competencies and/or objectives address each of the Roles in the CanMEDS/CanMEDS-FM Framework specific to the discipline.[32] (p. 9)<br>**3.2.2**: The residency program uses a comprehensive curriculum plan, which is specific to the discipline and addresses all the CanMEDS/CanMEDS-FM Roles<br>**3.2.2.3:** The curriculum plan addresses expert instruction and experiential learning opportunities for each of the CanMEDS/CanMEDS-FM Roles, with a variety of learning activities, including, but not limited to skills training, simulation, experiential learning, seminars, reflective exercises, directed reading….and others, as appropriate. (p. 10)<br>**3.2.4**: Residents' clinical responsibilities are assigned in a way that supports

colleagues, students, and co-workers from other disciplines;<br>3. Collaborator<br>3.1 Residents must be given opportunities to develop effective collaborative skills:<br>3.1.1 to work effectively with all members of the multi-disciplinary patient care team'<br>3.1.2 to consult with other physicians and health care professionals to provide optimal care of patients;<br>3.1.3 in conflict resolution (p.50-51)<br>**Standard B.6: Evaluation of Resident Performance**<br>2.5 Residents should be assessed for their performance, including interpersonal skills, in collaborating with all members of the patient care	the progressive acquisition of competencies and/or objectives, as outlined in the CanMEDS/CanMEDS-FM roles.<br>**3.2.4.3**: Residents' clinical responsibilities, including on-call duties, provide opportunities for progressive experiential learning, in accordance with all CanMEDS/CanMEDS-FM roles. (p. 11)<br>**3.4.1.5**: The system of assessment meets the requirements within the specific standards for the discipline, including the achievement of competencies in all CanMEDS/CanMEDS-FM roles and/or CFPC evaluation objectives (p.12)<br>**Domain: Resources**<br>**4.1.2.3**: Resident training takes place in functionally inter- and intra-professional learning environments that prepare residents for collaborative practice.

team and in the wise use of consultations with other professionals. (p. 51)	
**Nursing** - Canadian Association of Schools of Nursing (CASN)	
Source: Curran 2005, Table 1, page 39-40	Source: Canadian Association of Schools of **Nursing** (CASN) Accreditation Program Standards June, 2014[34]
**Accountability:**<br>6. The students learn that in order to achieve accountability to clients they need to demonstrate effective functioning in complex social systems (e.g. working collaboratively with the health care team; using socio-political skills; demonstrating behaviours associated with professional self-concept; participating in professional organizations).<br>**Relatedness:**<br>17. Situations for learning are representative of the	**Partnerships**<br>Standard Statement The educational unit is engaged in partnerships that support excellence in nursing education.<br>Descriptor Partnerships refer to collaborations that support the achievement of the unit's strategic goals, collaborations among educational units to deliver a collaborative nursing education program, and formal agreements with health service organizations, community-based agencies, members of other professions, and other relevant groups to provide professional and interprofessional learning opportunities for students. (P. 12)<br>**Teaching and Learning**

situations nurses encounter in practice (e.g. population groups; critical life events; illness problems; health profiles; institutional services; multi-disciplinary groups; autonomous practice).<br>Note: If site visit team determines that interprofessional education is important to a particular institution as a whole (i.e. through interviews with VP Academic, President of the university, and/or clinical partners), they will question the nursing school on this area.	Standard Statement Faculty members teaching in the educational unit foster excellence in nursing teaching<br>Key Element 7: Faculty are supported in providing interprofessional education and opportunities for intersectoral collaboration.* (P. 16)<br>*Intersectoral collaboration refers to collaboration with persons working in sectors of society other than health care.<br>**Environment**<br>Standard Statement The internal and external environments of the educational unit support excellence in nursing education.<br>Key Element 6: Practice placement sites provide learning opportunities that effectively help learners attain the outcomes of the nursing education program(s) and facilitate intra and interprofessional collaboration. (P. 18)<br>**Program Framework**<br>Standard Statement The program framework articulates a clear and coherent foundation for excellence in nursing education.

	Key Element 4: The curriculum….promotes interprofessional practice (P. 23)<br><br>Evidence includes:…examples of interprofessional education ….P. 24)<br><br>**Knowledge-based Practice**<br><br>Standard Statement Learners engage progressively in effective, knowledge-based practice.<br><br>Descriptor The program provides opportunities for learners to develop knowledge, skills, and attitudes ….in the delivery of collaborative client-centered care. (P. 25)<br><br>**Professional Growth**<br><br>Standard Statement Learners develop personally and professionally throughout the program<br><br>Descriptor The program provides opportunities for learners to develop the knowledge, skills, and attitudes to provide safe, ethical, and client-centred care as a member of the interprofessional team.<br><br>Key element 7: The program provides opportunities for students to develop functional

	working relationships, including intra/interprofessional and intersectoral collaboration. (P. 26)
**Occupational Therapy** - Canadian Association of Occupational Therapists (CAOT)	
Source: Curran 2005, Table 1, page 41-42	Source: CAOT Academic Accreditation Standards and Self-Study Guide, 2011.[35]
**Students are expected to:**<br><br>Increase their understanding of and respect the roles and functions of other team members;<br><br>Learn how occupational therapists contribute to the service delivery team.	**<u>Prepares graduates of entry level practice</u>**<br><br>2.3 The **educational conceptual framework** is based on current and emerging educational practice including interprofessional education<br><br>2.32 The **educational conceptual framework** incorporates new and emerging educational theories/practices/trends including interprofessional education (P. 17)<br><br>2.5 The **professional conceptual framework** is based on current and emerging occupation-based occupational therapy theory and practice including interprofessional practice (P. 18)

	2.71 A copy of the course outlines…….including interprofessional education<br><br>2.72 A report that documents and critiques the education methods…including interprofessional education and practice. (P. 19)<br><br>2.713 The **academic and fieldwork education** components incorporate interprofessional education and practice. (P. 21)<br><br>**Student Affairs services adequate**<br><br>3.11 **Resources** that convey information on the occupational therapy program, including practice, policies, regulations, resources, services and interprofessional education are accessible to all students, including students with special needs (P. 21)<br><br>**Environmental resources adequate**<br><br>6.2 There is sufficient **space** to support the occupational therapy program objectives, including interprofessional education.

	<u>**Learning resources adequate**</u><br><br>7.1 **Learning resources** are adequate to support the occupational therapy program objectives, including interprofessional education.<br><br>7.11 The **number and type of learning/instructional resources** are sufficient to carry out the occupational therapy program, including interprofessional education. (P. 31)<br><br>**Glossary**<br>Interprofessional education: Interprofessional education occurs when two or more professions learn with, from and about each other to improve collaboration and the quality of care. Students who participate in interprofessional education will develop and manage the knowledge, skills and attitudes necessary for effective client-centred collaborative practice. (p. 35)<br>**Glossary of Terms**<br>**Interprofessional Education**<br>Occasions when two or more professions learn with, from and about each other

	to improve collaboration and quality of care (CAIPE, 2002) IPE is a complex educational approach that is most effective when integrated throughout a program of study in both academic and practice learning as the student moves from simple to more complex learning activities. It is a critical emerging component of health professional education in Canada and internationally. (p. 37)
**Pharmacy** – Canadian Council for the Accreditation of Pharmacy Programs	
Source: Curran **2005,** Table 1, page 39-42	Source: Canadian Council for the Accreditation of Pharmacy Programs Accreditation Standards Guide **2018**[36]
**Guideline 5.2**<br>"The **University** should facilitate interprofessional health science education. Professional programs in pharmacy should benefit from association with other health science programs including shared didactic and clinical experiences and the utilization of	**Part 1: Academic Program (Standards 1-7)**<br>**Criterion 1.2** Students demonstrate practice-readiness that enables them to provide patient care as a collaborative member of a care team before starting culminating direct patient care required practice experiences.<br>**Standard 3:** The professional degree program in pharmacy has a minimum of four academic years..including…intraprofessional experiences that support

common physical and clinical facilities."<br><br>**Guideline 11.6**<br><br>"..Practice experiences should enhance communication skills with patients and other professionals, including the ability to work and communicate effectively with diverse Colleagues and patients."<br><br>**Guideline 12.2:**<br><br>"The curricular areas of pharmacy practice and structured pharmacy practice experiences should serve as the mainstay for the application and further development of interpersonal and interprofessional communicative and collaborative skills necessary to the rendering of pharmaceutical care."	educational outcomes.<br><br>**Criterion 3.3** The Faculty ensures that the professional program includes diversity of ….intra- and inter- professional educational experiences.<br><br>**Standard 4:** The curriculum includes foundational content in ….intra- and inter-professional collaborative practice skills.<br><br>**Standard 5:** …Experiences integrate, reinforce and advance the knowledge, skills, attitudes and values developed through other components of the professional program, including collaboration and teamwork.<br><br>**Standard 6:** The **curriculum** includes required intra- and interprofessional learning experiences, offered throughout the professional program, to enable a graduate to provide patient care as a collaborative member of a care team<br><br>**Criterion 6.1** Experiences address content to develop the expected competencies for intra- and interprofessional care and collaborative practice. Experiences are integrated throughout the professional program.<br><br>**Part 2: Governance and Program Management (Standards 8-22)**

	**Criterion 8.1** At a minimum, the other health sciences programs include Medicine and Nursing with whom the Faculty has effective collaborations. If the University does not have these programs, the Faculty has a formal alliance at another university within close proximity.<br>**Standard 11**: The **University** has integrated and endorsed the concept of interprofessional education and collaboration in practice.<br>**Criterion 11.1** The **University** enables relationships that support interprofessional learning.<br>**Criterion 11.2** Organizational structures and processes are in place to support interprofessional education.<br>**Criterion 11.3** Interprofessional Education is **recognized as a valuable teaching responsibility** within the academic health sciences.<br>**Standard 19**: Interprofessional education and collaborative practice is embedded in **Faculty policy and/or strategic plans**<br>**Criterion 19.1:** The policy and/or strategic plan includes the evaluation of

	interprofessional education.<br><br>**Part 3: Resources (Standards 23-30)**<br><br>**Criterion 25.3a** The Faculty works collaboratively with practice sites and other health professions programs to make intra- and/or inter-professional care/collaborative practice environments available to student pharmacists.
**Physical Therapy** – Physiotherapy Education Accreditation Canada (PEAC)	
**Source:** Curran 2005, Table 1, page 41	Source: PEAC Accreditation Standards, 2012.[37]
**6.5 Communication and Interdisciplinary Practice:**<br><br>Physiotherapy students, upon graduation will communicate with clients, relevant others and health team members to achieve interdisciplinary collaboration and service coordination by having knowledge and skills of:<br><br>6.5.1 Documenting relevant aspects of client history,	**Standard 4: Students**<br><br>**Criterion 4.2** Methods of teaching and learning align with program and curricular goals<br><br>**Examples of Evidence:** interprofessional learning opportunities (P. 20)<br><br>**Standard 6: Physiotherapy Competencies**<br><br>**Role 6.1 Expertise in Physiotherapy**<br><br>**Criterion 6.1.2** Analyze assessment findings, and establish a physiotherapy diagnosis and prognosis.

assessment, planning, intervention, discharge and follow-up<br>6.5.2 Effective written, verbal and non-verbal communication skills<br>6.5.3 Responsibility to refer to other physiotherapists and members of the health team when required<br>6.5.4 Providing education for clients and colleagues using pedagogical principles<br>6.5.5 Consulting and collaborating with individuals, other professionals, and community-based organizations to facilitate delivery of services<br>6.5.6 Informed consent and participatory decision-making	**Explanatory notes:**<br>Discuss physiotherapy diagnosis and prognosis with the client, and other health professionals / team members as appropriate.<br>**Criterion 6.1.3** Develop and recommend a physiotherapy intervention strategy. Implement the intervention<br>**Explanatory notes:**<br>Maintain continuity in physiotherapy service delivery, where resources permit (e.g. communicate with physiotherapists and other health professionals who share responsibility for service delivery, arranging for substitute service, as appropriate) (P. 28)<br>**Role 6.2 Communication**<br>**Criterion 6.2.1** Develop and maintain professional relationships through effective communication.<br>**Criterion 6.2.2** Gather and share information related to client care.<br>**Explanatory notes**

	Seek out and gather information from clients and other team members to assist in shared and informed decision-making<br>Communicate PT intervention strategies to team members effectively, and collaboratively set treatment priorities.<br>**Criterion 6.2.3** Employ effective and appropriate communication strategies. (P. 29)<br>**Role 6.3 Collaboration**<br>The program prepares students for collaborative practice to support quality client-centred care<br>**Criterion 6.3.1** Establish and maintain interprofessional relationships, which foster effective collaborative practice<br>**Explanatory notes:**<br>The program prepares students to:<br>• Demonstrate an understanding of and respect the roles, responsibilities, and differing perspectives of team members including clients.

	• Integrate knowledge and understanding of the physiotherapist role and the roles of others, including the client, in providing client-centred care.<br>• Consult and share relevant information with clients, other health professionals, and all relevant individuals or groups in a timely manner.<br>• Promote active and informed shared decision<br>**Criterion 6.3.2** Prevent, manage, and resolve conflict related to client-centred care<br>***Explanatory notes***<br>The program prepares students to:<br>• Demonstrate a respectful attitude towards colleagues and members of an interprofessional team, including clients.<br>• Identify the issues that may contribute to the development of conflict between the physiotherapist and client or among team members (e.g.,

	recognize how one's own beliefs, perceptions, and values may affect team dynamics and effectiveness).<br>• Address conflicts in an appropriate and timely manner. (P. 30)<br>**Role 6.5 Advocate**<br>**Criterion 6.5.1 Work collaboratively to identify, respond to, and promote the health needs and concerns of clients.**<br>**Explanatory notes:**<br>Collaborate with clients and other care providers to understand, identify, and promote the health and physiotherapy needs and concerns of clients. (p. 31)
**Social Work** - Canadian Association for Social Work Education - Association Canadienne pour la formation en travail social<br>Canadian Association of Social Work Education (CASWE-ACFTS)	
Source: Curran 2005, Table 1, page 40 and Curran 2008, Appendix E, pp. 94	Source: Canadian Association for Social Work Education (CASWE-ACFTS) Standards for Accreditation 2014[38]
	CLO #9 **Engage with individuals, families, groups, and communities through professional practice**<br>iv) Social work students are prepared for interprofessional practice,

	community collaboration and team work.
**SB 5.10.12:**<br><br>"The curriculum shall ensure that the student will have knowledge of other related occupations and professions sufficient to facilitate interprofessional collaboration and team work."	**Principle 2**<br><br>Social work education links together the interdisciplinary theoretical knowledge base of social work to professional practice.<br><br>**Domain 3. Program Content: Curriculum and Field Education**<br><br>3.1 Curriculum<br><br>3.1.9. iv) Social work students are prepared for interprofessional practice, community collaboration and team work.
