# Supplementary material for: Development of accreditation standards for interprofessional education: a Canadian Case Study
Source: Hum Resour Health. 2021 Jan 20;19:12. doi: 10.1186/s12960-020-00551-2 (PMC7818738; doi:10.1186/s12960-020-00551-2)
Supplement: Supplementary file 2 — Additional file 2. Narratives outlining progress in accreditation of IPE across the health/social service professions involved in AIPHE. [file 12960_2020_551_MOESM2_ESM.pdf]

Appendix 2

Narratives outlining progress in accreditation of IPE across the health/social service professions involved in AIPHE

Pharmacy:

In 2008 Pharmacy already had relatively robust IPE language in its standards. In 2012 and again in 2018 new standards were developed, sent out to stakeholders for consultation, and approved by the Board. The most recent IPE and IPC standards and evidence are even more explicit and comprehensive.[36] A big change involved using outcomes-based language that included achieving ‘team-readiness’. Within the ‘Academic Program’ category numerous standards and criteria make explicit reference to students needing to demonstrate collaborative competence through diverse practice and non-practice based interprofessional education opportunities along the learning continuum. Under the ‘Governance and Program Management’ category, separate standards require program to have close relationships with a network of health sciences programs, including, at a minimum, Nursing and Medicine. Standard 11 requires the University to recognize IPE as a valuable teaching responsibility, demonstrate organizational structures and processes that support IPE, and embed IPC and IPE in Faculty strategic documents. ‘Resources’ specify the need for resources to be allocated to developing practice based interprofessional education experiences.

Social Work

The 2014 CASWE-ACFTS Standards for Accreditation for undergraduate social work students (Bachelor of Social Work) and graduate social work students (Master of Social Work) refers to interprofessional education in two critical areas.[38] The first addresses IPE standards with respect to principles that guide the accreditation of social work programs in

Canada and emphasizes the link between knowledge and practice: "Principle 3. Social work education links together the interdisciplinary theoretical knowledge base of social work and professional practice." The second reference to IPE within the Standards for Accreditation states that curriculum content should include a core learning objective of professional practice with a specific requirement of the following "Social work students are prepared for interprofessional practice, community collaboration and team work."

Undergraduate Medicine

The accreditation of Canadian medical schools is done jointly by the Committee on Accreditation of Canadian Medical Schools (CACMS) with the Liaison Committee on Medical Education (LCME) in the USA using the same standards with some elements differing. At the time of the 2005 and 2008 baseline surveys, there were no standards that were specific to IPE or collaboration. In the most recent Standards and Elements document published in 2019, there are two standards under Curricular Content that speak to the importance of collaboration. Element 7.8 (Communication Skills) and Element 7.9 (Interprofessional Collaboration Skills) include specific language that ensures that students are prepared to function collaboratively on health care teams.[30]

Post Graduate Medical Education

Three organizations are responsible for the accreditation of all postgraduate residency programs in Canada: the College of Family Physicians of Canada (CFPC), the Royal College of Physicians and Surgeons of Canada (RC) and the Collège des médecins du Quebec (CMQ). All programs are transitioning to competency-based education models guided by the CanMeds framework.[32] One of the explicit roles with specific competencies is that of the "Collaborator". On July 1, 2018, a new set of conjoint generic standards were adopted across

all three organizations that were used to develop discipline-specific standards.[31,33] Standard 2 (Program Organization), Standard 3 (Educational Program) and Standard 4 (Resources) include a number of elements that refer to educational experiences that prepare residents for collaborative practice.

Nursing

In 2011, although there was existing language around 'partnerships' in the nursing program, there were no standards specific to IPE. An accreditation advisory committee was created in March 2011, to look at the issue of updating standards. In 2013 some minor changes incorporated IPE language into the existing standards guided by the AIPHE document. Key elements explicitly incorporated interprofessional education at the institutional, curriculum, and faculty levels. For example, the standard on teaching and learning stated, "faculty is supported in providing interprofessional education and opportunities for intersectoral collaboration." A major review was carried out in 2014 that resulted in further integration of IPE language and evidence and the revised program was formally adopted and introduced in May 2015.[34] In 2016 the accreditation program began to offer annual distance delivered workshops for faculty on IPE and a video was developed in 2017 for schools preparing for an accreditation review that provides them with information on implementing the expectations related to IPE specified in the accreditation standards.

Physical Therapy

In 2011, the profession underwent a major curriculum renewal. Its accreditation standards are largely dependent on national essential competencies for physical therapists in Canada which were recently revised based on the CanMEDS competencies model introduced initially by the medical profession. The AIPHE IPE principles were considered by the

national accreditation body and some of the terminology was embedded into the accreditation standards. In addition, the Physical Therapy Education Canada (PEAC) Standards Development Working Group was asked to obtain stakeholder input, and to test standard revisions to see if they were feasible. PEAC chose to embed IPE language throughout the 5 + 1 model of accreditation.[37] It is therefore integrated into the 6 domains of accreditation: organizational commitment, students, faculty unit, academic unit, educational program, and resources. Accreditation surveyors are required to examine the level and quality of IPE in all Canadian Physical Therapy programs. This same attention to IPE is now embedded in the new accreditation program for physical and occupational therapy assistant training programs in Canada.

Occupational Therapy

The Canadian Association of Occupational Therapists (CAOT) Academic Accreditation standards and indicators were revised in 2017 and another minor revision in 2019, replacing the earlier 2011 version, which already included indicators related to IPE and collaboration. In the latest version, however, the accreditation committee minimized the redundancies in providing evidence about IPE. In the revised standards, programs are still required to demonstrate IPE but the evidence is requested in fewer standards. They have also provided a definition of IPE in their glossary and updated the CAIPE reference in 2019.[35]
